# Remarkable Case of Hydrocephalus and Other Cerebral Disease in an Adult, with the Appearance on Dissection; and Some Observations on Epilepsy

**Published:** 1818-10-01

**Authors:** James Johnson


### *Remarkable Case of Hydrocephalus and other Cerebral Disease in an Adult, with the Appearance on Dissection; and some Observations on Epilepsy.*

By James Johnson, Esq.

THE subject of this paper (Lieut. Jeans) was an officer in His Majesty's service, about thirty years of age, whom I first saw in August 1815. I learnt that about two years previously, while at Alicant, in Spain, and during extremely hot weather, he had used very violent exercise in ascending a high mountain under a meridian sun; in consequence of which, he experienced a *coup de soliel.* A short time after this, he fell down in a fit at Carthagena, from which he was recovered by the abstraction of four pounds of blood, and very active purgatives. Previously to this period, he had been subject, for many years, to very profuse nasal hæmorrhages, which entirely ceased after the *coup de soliel* at Alicant. He was now observed to be gloomy in his mind, irritable in his temper, and to frequently complain of pain in the *left* side of his head, and about the occiput. These complaints forced him to relinquish active service, and return to England. Shortly after his arrival, he was observed to have convulsive twitchings in various parts of the body, which gradually increasing, ended in epileptic paroxysms. These were at first mild, transient, and at considerable intervals; but they soon gained strength, and returned at shorter but irregular periods. During the next eighteen months, he had been under the care of several medical gentlemen in succession, two of them experienced physicians. The pain in the head gradually increased with the violence of the epileptic seizures. His intellect became impaired; his temper grew dreadfully irritable, with great derangement of function in the chylopoietic organs, and some degree of fatuity after each paroxysm of epilepsy.

Under these medical gentlemen, he had been cupped, blistered, briskly and repeatedly purged. A seton had been established in the nape of the neck, and he had undergone a mercurial course with little, or at least temporary advantage.

On the 21st of August, 1815, or two years from the commencement of his illness, he was placed under my care; the following being the principal symptoms at this period, viz. Eyes very prominent, and somewhat suffused; countenance indicative of incipient fatuity, and great anguish, or at least suffering. The speech was stammering; the memory still more impaired than the judgment; since he answered questions pretty rationally and distinctly, but had great difficulty in recollecting names or terms. He complains of the most excruciating, but by no means continual pain in his head, especially on the left side and back part. The pupils are large, but contract freely. An occasional wild stare, loud idiotic laugh, instantly veering round to deep sighs, groans, and melancholy, proclaim the deranged state of the sensorium. Yet the pulse is natural; the tongue clean; the appetite good; the plumpness of body little diminished; but the stools are dark-coloured, and very offensive. His sleep is disturbed; temper extremely irritable. He has lately had an epileptic paroxysm after an immunity of nearly five months; and it was in consequence of this unexpected return of the convulsive attacks that medical assistance was called in. I stated to his friends that I apprehended such organic derangement had taken place in the head, as would entirely frustrate any remedial measures of mine; but I was importuned to take him under my care.

I could do nothing else than reiterate what had been before prescribed. He was cupped; took mercurial purgatives daily; was enjoined abstinence. After some time he took the tinct. lyttæ with the view of producing a determination of blood to the urinary organs. Under this treatment he seemed to amend; so much so, that he was able to walk a distance of three miles or more every day. But as winter approached, and the gloomy weather set in, the epileptic seizures became more frequent, and every paroxysm was succeeded by a longer and longer stage of mental fatuity, though the cephalea was almost invariably relieved for a time by the convulsive fit.

At length he tottered in his walk, and became confined within doors, while the head-aches were occasionally most excruciating. He now suffered severely from cramps and convulsive twitchings in different parts of the body, but particularly in the upper and lower extremities of the left side. He was also subject occasionally to involuntary discharges of urine and fæces.

About the beginning of July 1816, the pains in the head became periodical. During the day, while sitting below stairs, on a chair or sofa, he complained of no pain whatever; but was stupid, morose, and taciturn; his appetite continuing good, and his bowels constipated, unless when taking aperient medicines. After retiring to bed, he would sleep sound and easy till one, two, or three o'clock, when he would awake in such agony with the head-ache as to cause him to roar out and disturb not only the family, but the neighbourhood for three, four, or five hours; after which the pain would entirely cease till the next morning. The arsenical solution was now pushed as far as prudence would permit, without producing the smallest alteration, while blisters were kept open on the back of the neck for several days without effect. All this time the vascular system was perfectly tranquil, while the nervous system exhibited much derangement. Hemiplegia of the right side now took place, continued a few days, and then suddenly disappeared. Hyoscyamus, and even opium were now had recourse to, for the purpose of alleviating those morning paroxysms of agony, but with little or no effect.

On the 20th of July he ate heartily, spoke rationally, and walked up to bed himself at ten o'clock. He slept as soundly and tranquilly as ever he had done in his life, till two o'clock, when, as usual, he awoke in agonizing pain. His corporeal sufferings, however, were now near a close! He started up in bed, put his hand on his forehead, cried in a tremulous voice—"Oh, my head!" and instantly expired.

*Sectio Cadaveris, twenty-four Hours after Death, 22d July,* 1816.

Present, Dr. Denmark, Mr. Vowel, and myself.

The cranium was remarkably thin; in some places not thicker than a shilling. The dura mater adhered moderately to the skull, and exhibited nothing unusual on its external surface. It was readily raised from the subjacent pia mater of the *right* hemisphere, but both membranes were so intimately glued together, and to the substance of the *left* hemisphere, that they could not be separated from each other, nor from the brain, without tearing up the latter. The right hemisphere appeared pretty natural, but remarkably soft. The left hemisphere was of a browner colour, felt quite rotten, and as it were dissolved. On touching the left hemisphere, it trembled and undulated like a quagmire. The instant that the knife was aplied to make the *sectio ovalis* on this side, a stream of clear water gushed out to the distance of a foot from the wound, and continued to flow into the skull-cap till more than half a pint was collected.

The left lateral ventrical was then laid open, and was found to extend the whole length of the hemisphere, from os frontis to occiput. Its parietes were condensed and tough, even the septum lucidum was like a membrane in structure. Two ounces of water were sponged from the bottom of this ventricle. The foramen of Monro was half an inch in diameter. An ounce or two of water were taken from the right ventricle. The basis of the left anterior and middle lobes of the cerebrum adhered firmly to the dura mater, spread over the corresponding bones of the skull. The dura mater covering the petrous portion of the temporal bone on this side was greatly thickened by successive layers and depositions of coagulable lymph; and both it and the contiguous portion of brain round the auditory nerve, appeared in an incipient state of suppuration. The tentorium, especially over the left lobe of the cerebellum, was much thickened, diseased, and adherent to the cerebellum itself. The right lobe of the latter could be raised from its bed, but the left was obliged to be torn up piecemeal. An abscess was found between the two lobes of the cerebellum, and right along the inferior crucial spine of the occipital bone. The sac was an inch and a half long, by three quarters of an inch in breadth. Some yellow tenacious matter was contained in it. *The left lateral sinus was completely converted into, or filled up with, a solid body of a semicartilaginous texture and brown colour, and no trace of a canal left.* The dura mater about this part of the skull was, in some places, a quarter of an inch in thickness, and of a semicartilaginous hardness. The coverings of the medulla oblongata, and medulla spinalis, as far down as a slender knife would reach, were dreadfully diseased and thickened, with effusions of water.

---

REMARKS.

The phænomena of this disease and the *post mortem* appearances are calculated to afford matter for much useful reflection. The state of the dura mater exhibits the most unequivocal proofs that chronic, if not frequently *acute* inflammation, existed from the first attack at Alicant, three years before the fatal termination. The thickening of this membrane, in various places, to nearly a quarter of an inch, and its degree of induration nearly approaching to that of cartilage, evince the long duration of the inflammatory process. The obliteration of the left lateral sinus by a carneo-cartilaginous body, must also have commenced early [perhaps from the first attack, or *coup de soleil,* or a few months afterwards, when he fell down in a fit at Carthagena], and taken a long time to produce the present appearances. How long purulent matter had been formed between the cerebellum and its coverings, it is impossible to say ; but from the thickened state of the parietes of the abscess, and the inspissated appearance of the pus itself, owing, probably, to the thinner parts having been absorbed, we may assign to this event also a remote date. The distention of the lateral ventricle to such an enormous size, must have been a work of time; the process being so gradual, as to occasion few or none of those symptoms generally considered characteristic of hydrencephalus.

I think it highly probable, that the series of events was in the order above observed, and that the links were as *cause and effect* to each other. Thus the acute, and afterwards the chronic inflammation of the membranes, obliterated by successive depositions the left lateral sinus, and produced abscess in the cerebellum, whilst the great obstruction to the return of blood from the head, in consequence of half its channels being blocked up, may be fairly considered as the cause of the serous effusion in the ventricle of the same side.

I have yet said nothing of the symptoms resulting from such a slow, but terrible march of disorganization in so vital an organ as the brain. They offer a most important and memorable lesson to the medical practitioner! Had the cephalalgia which appeared at the beginning, and continued till the end, been early met by rigid abstinence from animal food and fermented liquors; by general and local blood-letting, repeated at longer or shorter intervals; by free evacuations from the bowels; by a slight and long continued mercurial action on the absorbent system ; by tranquillity of mind and body, what a tragic scene might have been averted!

How far the antiphlogistic plan was necessary or salutary after the effusion of water in the ventricles had commenced, I cannot presume to say. From a contemplation of the organic lesion by which it was preceded, if not occasioned, it would not appear likely that any mode of treatment, at *this period,* could do more than procrastinate the fatal issue.

The day was then past when any thing effectual could be attempted with any prospect of success. The symptom which most engaged the attention of the medical friends is now to be noticed—namely, the epilepsy.

This disease is so terrible in its appearance, inveterate in its nature, and deplorable in its consequences, that it has attracted the notice of all classes of society, in every age. In ancient times, as is well known, it was attributed to the ire of the gods or dæmons themselves. The schools teach us that epilepsy arises from two very opposite states of the brain, or, indeed, of the system altogether,—viz. inanition and repletion—In some cases from preternatural *irritability* of the nervous system. The effects of apparent inanition, however, have probably been much mistaken. In numerous experiments on animals, it has been proved that, when they are bled to death, [which must surely induce a pretty fair specimen of inanition] the vessels of the brain are generally found gorged with blood, and the ventricles full of water.* Here too convulsions close the scene; but whether these result from the extreme depletion of the general system, or *the local turgescence and effusion in the brain,* may admit of dispute. Reasoning from analogy in other cases, the convulsions are more attributable to the *latter* than to the former cause.

The organic disease giving origin to epilepsy can only be considered as the *predisposing cause,* otherwise its operation would be constant, which is not the case. There is every reason to believe, that the *proximate* cause is determined by a temporary turgescence of the vessels of the brain, or obstruction to its free circulation there: Hence, evacuations and low regimen have been found the best preventives of epilepsy, as was maintained by the late Dr. Fothergill, who in the 6th vol. of the Medical Observations and Inquiries, has given many excellent directions on this head.

The causes which induce these occasional determinations to the brain, are often beyond our recognition; but, that they *do* take place in every case of epilepsy was evidently the opinion of Dr. Fothergill—perhaps of Cullen; and certainly of Parry. But it may be said, if sanguineous turgescence in the vessels of the brain, produced or accompanied the epileptic paroxysm, how could some of the metallic salts, and many of the fœtid gums, have proved beneficial? It may be answered, that the laws which regulate the equal balance of the circulation, and the causes which disturb the equilibrium, are by no means

---

* *Vide* Dr. Seeds's interesting Inaugural Thesis in the first and fifth Nos. of the Medico-Chirurgical Journal.

clearly ascertained. If, as we have reason to believe is the case, the *nervous system* exercises an influence on the state of the *vascular system,* then the operation of the foregoing remedies may be more easily conceived. Their salutary effects, however, have been explained on another principle by Dr. Fothergill. "Valerian," says he, "castor, the fœtid gums, empyreumatic oils, and if there be any thing still more disgustful, commonly make a part of the medicines proposed for this disease. May not, therefore, these kinds of medicines, and most of those made use of as specifics from ancient authority, now and then confirmed with instances of benefit, derive the greatest part of their consequence from their *quantity,* or their *disgusting qualities,* which, by lessening the appetite, allow Nature to recover herself, and shake off a disease which indulgence principally produced." There may be something in this; and the argument is probably applicable to a great many other diseases, and other remedies.

The success which has lately attended the administration of certain stimulants, particularly the *lytta,* and oil of turpentine, instead of militating against the doctrine of *cerebral turgescence* and irritation, as the proximate cause of epilepsy, rather confirms it. These stimuli produce irritation in, and, (what is called) a determination of blood to, certain other organs and parts of the body—particularly the urinary apparatus and alimentary canal. It is during the continuance of this irritation, or determination to a distant part, that the encephalon obtains an immunity from disturbance.

I shall here relate an occurrence which happened in my own practice several years ago, which I think strongly illustrates this principle, though I then viewed it in a very different light, and drew, I am convinced, a most erroneous conclusion.

Happening to be in a situation in His Majesty's service, where a very considerable number of men were epileptic; but where, from particular causes, there was great temptation, and great inclination among these people to leave the service, I was naturally on my guard against imposition, and tried several means of detecting any simulated fits, but without success. In one case, however, I had much reason to suspect imposture, and I accordingly applied a blister from the nape of the neck to the sacrum, seven or eight inches in breadth, dressing the denuded surface with the cerat. lyttæ. The irritation of the blister, or absorption of the fly, produced a violent strangury with even bloody urine, and I began to fear I had carried my experiment too far; but I consoled myself with the idea of having detected my gentleman; for, whereas he heretofore seldom passed a day without an epileptic paroxysm, he did not now fall down once during the time that the blistered surface was raw, nor for more than two weeks after it was healed. I concluded that I had hit upon the right key, and accordingly blistered my epileptic patients most extensively on the back, in almost all of whom strangury took place, as in the first instance, and no one fell down while the back was raw, or any heat of urine remained. I pronounced them all impostors in my own mind, and my suspicions were apparently confirmed on finding them come back on the list, one by one, with the old complaint, in a few weeks after the blistering plan was left off. Provoked beyond measure, I blistered them more extensively and severely than ever, and with a result as before. Events occurred shortly after this, that separated me from my patients for ever; but I carried with me a conviction, that I had only checked imposture; whereas, I now firmly believe, that I had arrested, *pro tempore,* the attacks of real epilepsy.

Since that period I have administered the tinctura lyttæ in several cases with good effect. When the urinary organs came under the influence of the medicine, the paroxysms were generally moderated in force, and the intervals lengthened in duration. I have had some patients, who took as much as eighty or ninety drops twice or thrice a-day, without any other inconvenience than a trifling strangury. They always observed that these drops had a most exhilarating influence on their spirits. Indeed, I have lately found very beneficial effects result from the use of this remedy in certain derangements of the nervous system; which may, probably, be the subject of a future communication. I am strongly disposed to think, that in certain species of mania, it might be a powerful remedy.*

JAMES JOHNSON.

*London,* 1st *of September,* 1818.

---

* *See* Dr. Percival's Paper on Oil of Turpentine in Epileptic Mania, in the Analytical Department, and the ***Second Edition*** of my work on Atmospheric Influence, where I have introduced a Chapter on Epilepsy, and, I hope, brought forward some new and important views of the disease.

